# Keeping Cell Death in Check: Ubiquitylation-Dependent Control of TNFR1 and TLR Signaling

**DOI:** 10.3389/fcell.2019.00117

**Published:** 2019-06-28

**Authors:** Laura Griewahn, Aaron Köser, Ulrich Maurer

**Affiliations:** ^1^Institute of Molecular Medicine and Cell Research, University of Freiburg, Freiburg im Breisgau, Germany; ^2^Spemann Graduate School of Biology and Medicine, University of Freiburg, Freiburg im Breisgau, Germany; ^3^Faculty of Biology, University of Freiburg, Freiburg im Breisgau, Germany; ^4^BIOSS Centre for Biological Signalling Studies, Freiburg im Breisgau, Germany

**Keywords:** TLR, TNFR1, TNF, ubiquitin, inflammation, apoptosis, necroptosis

## Abstract

Pro-inflammatory signaling pathways, induced by pathogens, tissue damage or cytokines, depend on the ubiquitylation of various subunits of receptor signaling complexes, controlled by ubiquitin ligases and deubiquitinases. Ubiquitylation sets the stage for the activation of kinases within these receptor complexes, which ultimately regulate pro-inflammatory gene expression. The receptors, which transduce pro-inflammatory signals, can often induce cell death, which is controlled by ubiquitylation as well. In this review, we discuss the key role of ubiquitylation in pro-inflammatory signaling by TNFR1 and TLRs and its role in setting the threshold for cell death induced by these pro-inflammatory triggers.

## Building Up Signaling Complexes: Ubiquitylation in Immune Receptor Signaling

Inflammation is essential for the initial response to a pathogen or to tissue damage. If a pathogen overcomes barrier tissues such as the skin or the intestinal epithelium, an inflammatory response is induced by cells of the innate immune system, such as macrophages and dendritic cells. These cells, but also epithelial cells, endothelial cells as well as fibroblasts express germline-encoded pattern recognition receptors (PRR), which recognize structures or factors typical for pathogenic microbes. Structures associated with pathogens such as bacteria or viruses (but not the host), collectively dubbed as pathogen associated molecular patterns (PAMPS), are recognized by transmembrane TLRs and C-type lectin receptors (CLRs), as well as by cytoplasmic receptors such as the Retinoic acid-inducible gene (RIG)-I-like receptors (RLRs), DNA sensing receptors (DSRs), and NOD-like receptors (NLRs).

Likewise, molecules indicative of cell damage (danger associated molecular patterns, DAMPS) are recognized by TLRs or IL-1 family receptors (Takeuchi and Akira, 2010; Martin, 2016).

The engagement of PRRs ultimately induces the transcription of genes encoding cytokines, chemokines and interferons. PRR signaling requires ubiquitin ligases, generating ubiquitin chains with different linkage types. The pro-inflammatory cytokines induced by PRR activation, such as TNF or IL-1, again heavily depend on poly-ubiquitylation for the signaling pathways they induce in target cells and tissues. As described in this article, ubiquitylation also has a key role in preventing cell death induced by these triggers.

Ubiquitylation is a posttranslational protein modification by which ubiquitin, a small protein, is reversibly linked to protein substrates. Ubiquitin ligases enzymatically link the C-terminus of ubiquitin, a small, 8 kD protein, to the ε-amino group of a lysine of a given protein, including ubiquitin itself. In addition, ubiquitin can be linked to the free amino group of the methionine of another ubiquitin. This energy-requiring transfer is accomplished in three separate steps. Firstly, upon ATP consumption, a free ubiquitin is transferred to a cysteine of an ubiquitin-activating enzyme, forming a high-energy thioester (E1). Ubiquitin is then transferred from E1 to an E2 enzyme. The human genome encodes only two E1 ligases, but about fourty E2 conjugating enzymes, which exhibit a first level of specificity. The transfer of ubiquitin from E2 to the target protein is mediated by one of several hundred different substrate-specific E3 ligases. E3 ubiquitin ligases can mediate the attachment of either single ubiquitins to target proteins, resulting in protein mono-ubiquitination, or using an attached ubiquitin as an anchor to generate extended polyubiquitin chains (Ebner et al., 2017). The primary structure of ubiquitin contains seven lysines (K6, K11, K27, K29, K33, K48, and K63), the ε-amino group of which can be linked to the C-terminus of an incoming ubiquitin, thereby creating an isopeptide bond. In addition, the free amino-terminus of ubiquitin (M1) can be linked with the C-terminus of another ubiquitin, resulting in a peptide bond, which generating M1-linked ubiquitin chains. Thus, depending on the linkage specificity of the respective E3 ubiquitin ligase, poly-ubiquitin chains with different inter-ubiquitin linkages can be generated (Komander and Rape, 2012; Akutsu et al., 2016).

Ubiquitylation is a reversible posttranslational modification and poly-ubiquitin chains are disassembled by ubiquitin-specific proteases. These deubiquitinases, just as ubiquitin ligases, exhibit substrate specificity with regard to the linkage of the ubiquitin bond they hydrolyze. About one-hundred deubiquitinases exist in humans, which attenuate or erase the signal mediated by ubiquitin ligases (Mevissen and Komander, 2017).

Poly-ubiquitin chains exhibit their function through their recognition by proteins containing ubiquitin binding domains (UBDs), which bind and thereby ’read’ those structures (Dikic et al., 2009). UBDs are specific for the structure of ubiquitin chains, depending on the linkage type, or they recognize linker regions directly, and their affinity may depend on the length of the ubiquitin chain (Rahighi and Dikic, 2012). In analogy to chromatin modifiers in epigenetics, ubiquitin ligases can be considered as “writers” and UBDs as “readers.” Accordingly, deubiquitinases function as “erasers” (Komander and Rape, 2012).

The recognition of ubiquitylation by UBDs triggers diverse biological processes. The attachment of K48-linked polyubiquitin chains to a protein is a signal for its degradation by the proteasome. K63- and M1-linked polyubiquitin chains have various functions, such as in DNA repair or for the activation of kinases in receptor complexes (Ulrich and Walden, 2010; Jiang and Chen, 2011). The role of K6-, K11-, K27-, K29-, and K33-linked ubiquitylation is comparably less well understood and reviewed elsewhere (Akutsu et al., 2016).

For both TLR and TNF signaling, K63- and M1-linked polyubiquitination is essential for the formation of the signaling complexes, which ultimately mediate NF-κB and MAPK activity and pro-inflammatory gene activation. As a general principle, the stimulation of an innate immune receptor induces the recruitment (via different adaptors) of E3-ligases (such as TRAF6 or cIAP1/2), which conjugate proteins in the receptor complex with K63-linked polyubiquitin chains. Adapter proteins which recognize these chains mediate the recruitment of the kinase TAK1, the activity of which is central to TLR and TNF signaling (Jiang and Chen, 2011). In addition, K63-linked polyubiquitin chains recruit the LUBAC complex, an M1-linkage specific ubiquitin ligase, to the receptor complex. LUBAC decorates proteins in the complex with M1-linked polyubiquitin chains, often by extending K63-linked with M1-linked polyubiquitin chains (Cohen and Strickson, 2017). This promotes the recruitment of the IKK complex, which comprises the kinases IKKα, IKKβ and the adapter NEMO/IKKγ, to the receptor complex, via the interaction of its subunit NEMO with M1-linked polyubiquitin chains. Being in the proximity of TAK1, IKKs are activated by TAK1 through direct phosphorylation. Once activated, the IKKs phosphorylate IκBα, which triggers its K48-linked polyubiquitylation and degradation, thereby liberating NF-κB transcription factors. In addition, TAK1, by phosphorylation of MKKs, activates p38 and JNK signaling, and thereby AP-1 transcription factor activity (Hrdinka and Gyrd-Hansen, 2017). Ubiquitylation in these receptor complexes, is counteracted by deubiquitinases, including CYLD, which exhibits linkage specificity for K63-and M1-linked ubiquitin chains, and OTULIN, which specifically disassembles M1-linked ubiquitin chains (Mevissen and Komander, 2017).

### TNFR1 Signaling Controlled by Ubiquitylation

Induction of PRRs result in the induction of various pro-inflammatory cytokines, and a crucial player among those is TNF. TNF is made by macrophages, monocytes, dendritic cells as well as activated lymphocytes, but also by non-professional immune cells such as epithelial and endothelial cells (Takeuchi and Akira, 2010). TNF-induced inflammation is beneficial in containing pathogens, but also has a key role for chronic inflammatory diseases. In consequence, TNF-inhibitory molecules proved to be successful for the treatment of inflammatory diseases, such as rheumatoid arthritis or psoriasis (Udalova et al., 2016).

There are two receptors for TNF. While TNFR1 is expressed ubiquitously, expression of TNFR2 is restricted to immune cells and endothelia (Wajant et al., 2003). In general, TNF triggers inflammation in tissues by inducing pro-inflammatory gene expression in target cells. It does so by induction of NF-κB and MAPK, which are activated through ubiquitylation-dependent signaling complexes. Upon ligation of the TNFR1, TNFR1 complex I is formed by the interaction of the death domain (DD) of the receptor with the DDs of the kinase RIPK1 and the adaptor TRADD, thereby independently recruiting both proteins to the receptor. TRADD recruits the protein TRAF2 and thereby the E3 ligases cIAP1/2 which decorate RIPK with K63-linked polyubiquitin chains (Bertrand et al., 2008; Varfolomeev et al., 2008).

Both free and attached K63-linked polyubiquitin chains have been reported to activate the kinase TAK1 (Wang et al., 2001; Xia et al., 2009). This is mediated by adaptor proteins (TAB2 and TAB3), which recognize K63-linked poly-ubiquitin chains (but not M1-linked polyubiquitin chains) via their zinc-finger UBD, activating the kinase TAK1 (Wang et al., 2001; Kanayama et al., 2004; Kulathu et al., 2009; Xia et al., 2009). TAK1 activation is a key event for pro-inflammatory gene expression, as TAK1 activates the IKK complex, through IKKβ phosphorylation, and MAPK signaling by the phosphorylation of MKK3, MKK6, and MKK4 (Moriguchi et al., 1996; Shirakabe et al., 1997; Lee et al., 2000; Wang et al., 2001).

However, full activation of the IKK complex requires the activity of another ubiquitin ligase, the LUBAC complex. LUBAC consists of the enzymatic subunit HOIP and the proteins HOIL-1 and SHARPIN and is the only identified E3 ligase capable of generating M1-linked polyubiquitin chains. K63-linked polyubiquitylation is the prerequisite for the association of LUBAC to the receptor complex, as the absence of cIAP1/2 abrogated the recruitment of LUBAC to the TNFR1 signaling complex (TNFR1-SC) (Haas et al., 2009). LUBAC was shown to interact with polyubiquitin via the Npl4 zinc finger (NZF) domains of HOIP and of HOIL-1 (Haas et al., 2009; Peltzer et al., 2018). Once associated with the TNFR1-SC, LUBAC was demonstrated to attach M1-linked polyubiquitin chains to RIPK1, TRADD and TNFR1 itself (Gerlach et al., 2011; Draber et al., 2015). There is convincing evidence that, upon TNF stimulation, LUBAC extends preexisting K63-linked poly-ubiquitin chains in the TNFR1-SC with M1-linked polyubiquitin chains, and those hybrid chains were attached to RIPK1 upon TNFR1 stimulation (Emmerich et al., 2016). In addition, TNFR1 itself is decorated with M1-linked polyubiquitin chains, possibly attached to multi-monoubiquitinated TNFR1.

M1-linked polyubiquitin chains are bound with high affinity by the IKK complex member NEMO, through its UBAN (ubiquitin-binding domain present in ABINs and NEMO), thereby mediating the association of the IKK complex with the TNFR1-SC (Lo et al., 2009; Rahighi et al., 2009). NEMO itself was shown to be subject to M1-linked polyubiquitylation by LUBAC (Tokunaga et al., 2009, 2011; Gerlach et al., 2011), while it was subsequently demonstrated that M1-linked ubiquitylation of NEMO is comparably low, questioning its relevance for signaling (Clark et al., 2013; Emmerich et al., 2013). The interaction of NEMO with K63-/M1-linked hybrid chains might bring the IKK complex, recruited to M1-linked polyubiquitin sections, into proximity of the TAB/TAK1 complex, associated with K63-linked polyubiquitin chain segments, facilitating the activating phosphorylation of the IKKs by TAK1 (Wang et al., 2001; Zhang et al., 2014; Cohen and Strickson, 2017). The activated IKK complex phosphorylates the protein IκBα, which triggers the K48-linked polyubiquitylation and degradation. Freed from IκBα, a NF-κB dimer can enter the nucleus and promote the transcription of genes promoting inflammation. MKKs activated by TAK1 induce the MAP Kinases JNK and p38 and activate the transcription factor AP1, which also induces the transcription of pro-inflammatory cytokines (Moriguchi et al., 1996; Shirakabe et al., 1997; Wang et al., 2001).

In cells stimulated by TNF, this transcriptional program induces a large number of genes, which bring about the changes typical for inflamed tissues. In addition, TNF ubiquitylation-dependently induces genes, which promote cell survival such as c-FLIP, as will be described in detail below.

**FIGURE 1:**
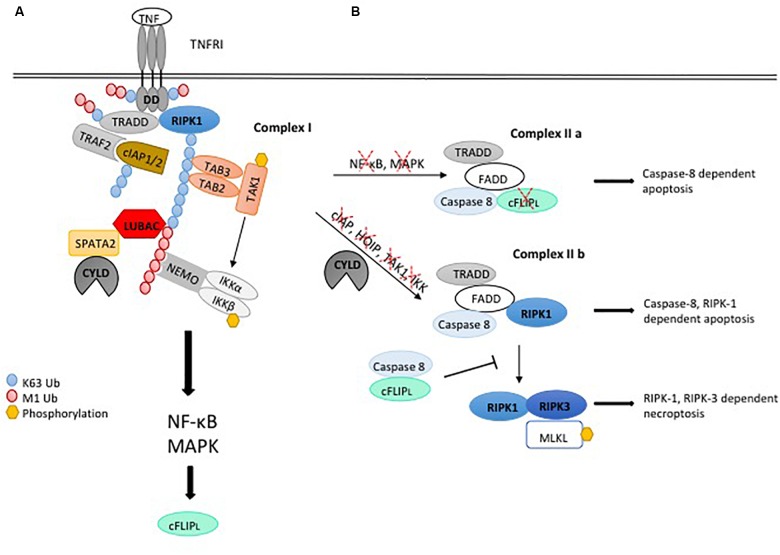
Ubiquitylation regulating pro-inflammatory signaling and cell death induction by the TNFR1-SC. **(A)** Upon binding of TNF to TNFRI, complex I is assembled by binding of the adaptor protein TRADD and the kinase RIPK1 to the receptor. Recruitment of TRAF2 and the E3 ligase cIAP1/2 leads to K63-linked ubiquitylation within the complex, serving as a platform for binding of TAB2/3 and TAK1 to mediate activation of MAPK signaling. In addition, K63-linked poly-ubiquitin chains recruit LUBAC, which mediates M1-linked ubiquitylation of different components within the complex, promoting association of NEMO and activation of IKKaα/β and NF-κB. Assembly of the TNFR1-SC is negatively regulated by the DUB CYLD, which interacts with the LUBAC component HOIP via the bridging protein SPATA2 and is recruited to the TNFR1-SC, thereby diminishing M1- and K63-linked ubiquitin chains. **(B)** TNFR1 stimulation induces functional variants of the cell death promoting complex II, consisting of the adaptor TRADD and FADD, RIPK1, caspase-8 and its paralog c-FLIP. Abrogation of NF-κB induced gene expression permits caspase-8 activation in complex II a, leading to cell death by apoptosis. When ubiquitylation of the TNFR1-SC is compromised, caspase-8-activating complex II b is formed, which induces apoptosis depending on RIPK1 kinase activity. Upon loss of caspase activity, RIPK1 associates with RIPK3, which phosphorylates and activates MLKL, leading to necroptotic cell death.

The disassembly of ubiquitin chains by DUBs has a crucial role in the regulation of TNFR1 signaling, either by mediating destabilization of the receptor complex, attenuating the signal, or by trimming/editing polyubiquitin chains. The K63-and M1-linkage specific DUB CYLD was shown to negatively regulate TNF-induced NF-κB and MAPK activation (Brummelkamp et al., 2003; Kovalenko et al., 2003; Trompouki et al., 2003; Lee et al., 2013). CYLD is recruited to the TNFR1-SC along with the LUBAC complex (Takiuchi et al., 2014; Draber et al., 2015; Hrdinka et al., 2016). This requires the adaptor protein SPATA2, bridging the interaction between CYLD and HOIP (Elliott et al., 2016; Kupka et al., 2016; Schlicher et al., 2016; Wagner et al., 2016; Figure 1A). CYLD was shown to counteract M1- and K63-linked ubiquitylation of RIPK1, TRADD and TNFR1 (Draber et al., 2015; Hrdinka et al., 2016). Consistently, the absence of SPATA2 was shown to exhibit increased M1-ubiquitylation in the TNFR1-SC (Schlicher et al., 2016). However, a reduction of RIPK1 ubiquitylation as well as K63- and M1-linked polyubiquitin was observed in cells lacking SPATA2 by other studies (Draber et al., 2015; Schlicher et al., 2016; Wei et al., 2017). While the reasons for those inconsistencies are not clear, different effects of CYLD and SPATA2 on ubiquitylation in the TNFR1-SC may hint at yet unidentified SPATA2 functions, which may not be directly linked to CYLD.

OTULIN specifically degrades M1-linked polyubiquitin chains, generated by LUBAC (Keusekotten et al., 2013; Rivkin et al., 2013). Just like SPATA2, OTULIN interacts with LUBAC through a PUB interacting motif (PIM), which associates with the PUB domain of HOIP, implying a competition of SPATA2 and OTULIN for binding to HOIP (Elliott et al., 2014, 2016; Schaeffer et al., 2014; Takiuchi et al., 2014; Schlicher et al., 2016; Wagner et al., 2016). However, in contrast to SPATA2 and CYLD, OTULIN was not found to be recruited with LUBAC to the TNFR1-SC upon TNFR1 stimulation. Accordingly, the absence of OTULIN did not affect M1-linked polyubiquitylation at the TNFR1-SC (Draber et al., 2015). However, different studies indeed detected OTULIN in TNFR1-SC pulldowns (Schaeffer et al., 2014; Wagner et al., 2016), and it was also shown that OTULIN disassembles M1-linked ubiquitin in receptor complexes (Fiil et al., 2013; Keusekotten et al., 2013). This is compatible with a concept that OTULIN functionally counteracts LUBAC, thereby limiting the activation of NF-κB and MAPK signaling. Consistently, mice with acute ablation of OTULIN in bone marrow cells or myeloid cells exhibited massive TNF-dependent systemic or chronic inflammation, respectively, reflecting patients with defective OTULIN, which exhibit multi-organ inflammation (Damgaard et al., 2016).

This view was challenged recently. It had been observed previously that LUBAC ubiquitylates itself, and that OTULIN deubiquitylates LUBAC components (Fiil et al., 2013; Keusekotten et al., 2013; Elliott et al., 2014; Draber et al., 2015; Hrdinka et al., 2016). In a recent study, knock-in MEF expressing catalytic-inactive OTULIN exhibited enhanced LUBAC auto-ubiquitylation with reduced abundance of HOIL-1 HOIP and SHARPIN. The authors reported reduced stability of TNFR1 complex I and enhanced formation of TNFR1 complex II in these cells (Heger et al., 2018). These data suggest that OTULIN promotes LUBAC activity and pro-inflammatory signaling, while preventing cell death, as described further below.

A third DUB implicated in TNFR1 signaling, A20, was recently shown to exhibit its function independently of its enzymatic activity. The recruitment of A20 to the TNFR1-SC required M1-polyubiquitin chains and actually resulted in protection of these chains from degradation. This was shown to depend on the ZnF7 zinc-finger domain of A20 providing the interaction with polyubiquitin chains and possibly shielding them from binding proteins activating gene expression (Nishimasu et al., 2012; Verhelst et al., 2012; Draber et al., 2015). Consistent with a minor role of A20 enzymatic activity, mice expressing DUB-inactive A20 mutant exhibit normal TNF-induced NF-κB signaling (De et al., 2014).

### TLR Signaling Controlled by Ubiquitylation

Pattern recognition receptors of the toll-like-receptor (TLR) family are germline-encoded and expressed by professional innate immune cells such as macrophages, monocytes and dendritic cells, but also epithelial cells, endothelial cells and fibroblasts. They recognize, through their leucine-rich ectodomains, an array of bacterial or viral structures, including lipids, proteins and nucleic acids. TLRs are located either on the plasma membrane or on endosomes and recognize bacterial patterns such as peptidoglycan through the TLR1/2 heterodimer, LPS through TLR4, flagellin through TLR5, and CpG DNA is detected by TLR9. Likewise, viral, double-stranded RNA is recognized by endosomal TLR3 (Takeuchi and Akira, 2010).

Toll-like receptors signal through two different pathways, depending on whether MyD88 or TRIF is recruited as an adapter to the receptor. Signaling of all TLRs except TLR3 requires the adapter protein MyD88, while signaling by TLR3 depends on TRIF. TLR4 is the only TLR, which signals via both MyD88- and TRIF-dependent pathways (Yamamoto et al., 2003a).

The MyD88-dependent pathway is also employed by receptors for cytokines of the IL-1 family (O’Neill, 2008). TLRs and IL-1 receptor share the Toll -and IL-1 (TIR) domain, which interacts with the TIR domain of the adapter MyD88 upon receptor activation. In turn, the DD of MyD88 recruits, through a homotypic interaction, the kinase IRAK4 via its DD, which promotes the additional recruitment of the kinases IRAK1 and IRAK2. The IRAKs now dissociate from the receptor complex and interact with TRAF6, an E3 ubiquitin ligase (Takeuchi and Akira, 2010). By cooperating with the E2 ligases Ubc13 and Uev1A, TRAF6 auto-ubiquitylates, but also generates K63-linked poly-ubiquitin chains which were shown to be attached to IRAK1, IRAK4, and MyD88 (Emmerich et al., 2013). In addition, IRAK1 and IRAK4 can phosphorylate the E3 ligase Pellino1, which also generates K63-linked poly-ubiquitin chains (Ordureau et al., 2008). Those protein-anchored and/or free K63-linked polyubiquitin chains provide the docking sites for the zinc-finger UBD of the adaptors TAB2/3, which bring the kinase TAK1 to the complex and activate it, thereby mediating activation of MAPK signaling (Wang et al., 2001; Kanayama et al., 2004; Kulathu et al., 2009; Xia et al., 2009).

**FIGURE 2:**
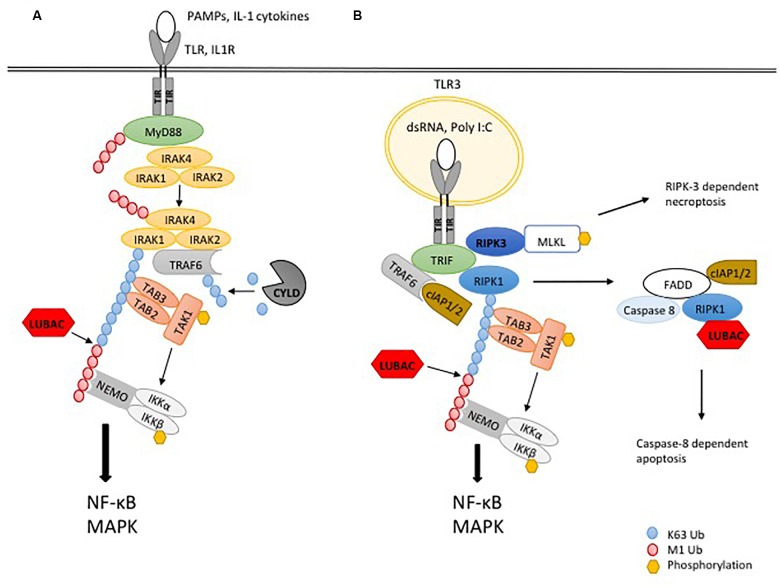
Ubiquitylation in TLR signaling. **(A)** The recognition of PAMPs or IL-1α/β by the respective TLR or IL-1R leads to binding of the adaptor protein MyD88, which interacts with the TIR domain of the receptor, leading to recruitment of the kinases IRAK4, IRAK1 and IRAK2. Dissociation of IRAK1 and IRAK2 and their interaction with the ubiquitin ligase TRAF6 initiates the formation of K63-linked poly-ubiquitin chains, serving as a platform for recruitment of TAB2 and TAB3 to activate the kinase TAK1 and in turn MAPK signaling. In addition, the ubiquitin ligase LUBAC extends K63-linked poly-ubiquitin chains with M1-linked poly-ubiquitin chains, resulting in recruitment and activation of the IKK complex through its adaptor NEMO. Phosphorylation of IKKβ by TAK1 leads to activation of NF-κB and pro-inflammatory signaling. The DUB CYLD counteracts ubiquitylation by TRAF6. **(B)** TLR3 signaling works independently of MyD88 but requires the adaptor TRIF. After binding of dsRNA or Poly I:C to the receptor, the kinase RIPK1 is recruited. The ubiquitin ligases TRAF6 and cIAP1/2 generate K63-linked poly-ubiquitin chains to activate TAK1 and downstream MAPK. To induce the activation of IKKs and NF-κB, LUBAC is recruited to the complex to generate M1-linked poly-ubiquitin chains. If K63- and/or M1-ubiquitylation are blocked, apoptosis is induced by a complex including FADD, caspase-8 and RIPK1.

As for TNFR1 signaling, LUBAC is required for full activation of NF-κB by the MyD88-dependent pathway (Cohen and Strickson, 2017). In further similarity to the TNFR1-SC, LUBAC extends preexisting K63-linked poly-ubiquitin chains in the complex with M1-linked polyubiquitin chains (Emmerich et al., 2013). M1-linked polyubiquitin chains, recognized by NEMO, promote the activation of the IKK complex (Lo et al., 2009; Rahighi et al., 2009), by a mechanism which was demonstrated to depend on TAK1-mediated phosphorylation and IKK auto-phosphorylation (Zhang et al., 2014; Figure 2A).

A different TLR signaling pathway, which is independent from the adaptor MyD88, instead requires the adapter protein TRIF (Yamamoto et al., 2003a). This pathway is employed by TLR3 and TLR4 upon binding of their ligands double-stranded RNA and LPS, respectively. TLR4 additionally requires the adaptor TRAM for the interaction with TRIF (Yamamoto et al., 2003b). TRIF is recruited to TLR3 or TLR4 through its TIR domain, interacting with the TIR domain of the activated receptor. Via its RHIM domain, TRIF recruits the kinase RIPK1 through a homotypic RHIM:RHIM interaction to the receptor, which is required for downstream NF-κB signaling (Meylan et al., 2004; Cusson-Hermance et al., 2005). The further assembly of the receptor complex is mediated by ubiquitylation, by the TRIF-mediated recruitment of the E3-ligases TRAF6 and cIAP1/2. These E3 ligases generate K63-linked polyubiquitin chains, activating the kinase TAK1, which in turn activates IKK and MAPK as described above (Cusson-Hermance et al., 2005; Shim, 2005). In addition, M1-linked ubiquitylation mediated by LUBAC was recently shown to be essential for TLR3 signaling. LUBAC was demonstrated to be recruited to activated TLR3, generating M1-linked polyubiquitin chains, which was essential for the activity of IKKs and MAPKs and the transcriptional induction of TNF or IL-8, as well as secretion of IFN-β. Interestingly, this study showed that the TLR3 receptor SC contains FADD and caspase-8 (Zinngrebe et al., 2016). As in the case of MyD88-mediated signaling, the presence of hybrid K63/M1-linked polyubiquitin chains was observed upon stimulation of TLR3, some of which were attached to RIPK1, while most of these chains were not linked to the kinase (Emmerich et al., 2016; Figure 2B). In addition to NF-κB and MAPK/AP-1 activation, TRIF mediates the induction of IFN-α and IFN-β, through phosphorylation of the transcription factors IRF-3 and IRF-7 by the IKK-related kinases TBK-1 and IKKε (Häcker and Karin, 2006). This requires the recruitment to TLR3 of the E3 ligase TRAF3 and its auto-ubiquitylation, resulting in further recruitment of the adaptors TANK, NAP1, and SINTBAD, which in turn recruit TBK-1 and IKKε to the receptor complex (Takeuchi and Akira, 2010).

The role and the regulation of deubiquitylases (DUBs) in MyD88 and TRIF dependent signaling is less well explored. In the MyD88 dependent pathway, CYLD was shown to reduce TLR2-mediated TRAF6 ubiquitylation and NF-κB activation (Kovalenko et al., 2003; Trompouki et al., 2003; Yoshida et al., 2005; Lim et al., 2007; Komander et al., 2009; Ritorto et al., 2014). More recently, CYLD was shown to reduce K63-linked polyubiquitylation of MyD88, thereby limiting MyD88-dependent cytokine induction and inflammation *in vivo* (Lee et al., 2016). This is consistent with data generated with CYLD deficient BMDM, which were shown to exhibit increased JNK activation upon stimulation with LPS (Zhang et al., 2006). In line with the role of SPATA2 being required for CYLD activity in receptor complexes, SPATA2 deficiency resulted in increased JNK signaling and cytokine expression of BMDM treated with LPS (Wei et al., 2017). However, it is noteworthy that another study did not find that the absence of CYLD in BMDM affected LPS-induced NF-κB and MAPK signaling (Reiley et al., 2006).

Together, the generation of ubiquitin chains is key for the induction of pro-inflammatory gene expression by TNFR1, IL-1R, and TLRs. This is reflected by genetic defects in humans, which affect M1-linked ubiquitylation, with severe pathologic consequences. Deficiency in HOIL-1 resulted in invasive pyogenic bacterial infection, likely due to an impaired induction of NF-κB, but also in autoinflammation and glycogen storage disease (Boisson et al., 2012). Similar defects were reported for a patient with a hypomorphic HOIP mutation (Boisson et al., 2015). Of note, the respective defects resulted in a destabilization of all the LUBAC components in these patients. While the observed pathologies can be explained by the inability to raise a pro-inflammatory response, an alternative explanation could be an enhanced susceptibility for cell death, as described below.

## Unleashing the Cell Death Machinery: Regulation by (De-)Ubiquitination

### Cell Death Induced by TNFR1

The default outcome of the signaling pathways described above is the induction of transcriptional programs, which regulate inflammation. However, upon certain conditions, inflammatory triggers can result in the induction of cell death, and the regulation of ubiquitylation is central in the decision for or against cell death. The predominant forms of cell death induced by immune/cytokine receptors are apoptosis and necroptosis. Both forms of cell death are triggered by the formation of protein complexes, which provide the platforms to activate the proteolytic activity of caspase-8 or the kinase activity of RIPK3.

TNFR1-induced apoptosis requires the activation of caspase-8 by homodimerization, which results in the cleavage and thereby activation of executioner caspases-3/-7 (Boatright et al., 2003). Caspase activation triggers a controlled form of cell death, leaving the plasma membrane intact and surrounding cells undisturbed. Thus, apoptosis is in general not expected to be pro-inflammatory or immunogenic.

In contrast, necroptosis requires RIPK3 activation by auto-phosphorylation, which is induced by dimerization via its RHIM and kinase domains (Cho et al., 2009; He et al., 2009; Raju et al., 2018). RIPK3 phosphorylates and activates the pseudokinase MLKL, which mediates Ca^2+^ influx and plasma membrane rupture (Sun et al., 2012; Cai, 2013; Khan et al., 2014). Necroptosis is morphologically indistinguishable from uncontrolled necrosis, with spillage of cytoplasmic contents into the environment of a dying cell (Zhang et al., 2016).

Some 20 years ago it had been observed that necrotic cell death induced by TNF occurs in absence of caspase activity (Vercammen et al., 1998). TNF-induced programmed necrosis is indeed repressed by the proteolytic activity of caspase-8, which is functionally separate from the apoptosis-inducing caspase-8 activity, exhibiting a different substrate specificity (Pop et al., 2011). Upon heterodimerization with cFLIP_L_, caspase-8 does not induce the activation of executioner caspases and apoptosis, but instead cleaves pro-necroptotic proteins such as RIPK1, RIPK3, and CYLD (Feng et al., 2007; O’Donnell et al., 2011; Oberst et al., 2011; Zhang et al., 2019). This pro-survival caspase-8 activity is the reason for the mid-gestation lethality of caspase-8 deficient mice, which was rescued in mice expressing a cleavage-deficient caspase-8 allele (which cannot undergo processing to its pro-apoptototic form) (Varfolomeev et al., 1998; Kang et al., 2008). The rescue of caspase-8 knockout mice upon additional loss of RIPK3 or MLKL provided genetic evidence for the inhibition of necroptosis by caspase-8 (Kaiser et al., 2011; Oberst et al., 2011; Alvarez-Diaz et al., 2016).

TNFR1 stimulation induces cell death via a signaling complex, which is different from the TNFR1-SC described above and therefore dubbed complex II. This complex is not associated with the receptor and comprises RIPK1, the adaptors TRADD and FADD, the initiator caspase-8 as well as the caspase-8 paralog c-FLIP (Micheau and Tschopp, 2003). It constitutes the platform to activate caspase-8 by induced proximity. However, as mentioned above, cell death upon TNFR1 stimulation is not the default outcome, because the TNFR1-SC transcriptionally induces the expression of pro-survival molecules such as c-IAP2 and c-FLIP, the latter coming in two splice forms, c-FLIP_s_ and c-FLIP_L_ (Chu et al., 1997; Micheau et al., 2001). The cFLIP_S/L_ molecules heterodimerize with caspase-8 and thereby inhibit the pro-apoptotic activity of caspase-8 (Hughes et al., 2016). Thus, TNFR1-SC signaling activates a transcription-dependent anti-apoptotic checkpoint, by the transcriptional induction of pro-survival proteins, preventing pro-apoptotic caspase-8 activation (Figure 1B).

Thus, any disturbance of the TNFR1-SC, interfering with M1- or K63-linked polyubiquitination, and/or the activity of the kinases, which require polyubiquitin chains for their activation, will reduce or abrogate the transcription-inducing activity of NF-κB and MAPK. Thereby, expression of pro-survival proteins such as cFLIP is compromised, allowing caspase-8 homodimerization in a complex, which is defined as TNFR1 complex IIa in this context. Many pathogens interfere with and subvert pro-inflammatory signaling [reviewed in Reddick and Alto (2014)], which may accordingly result in cell death. This checkpoint can be experimentally suppressed by inhibitors of transcription or translation such as actinomycin D or cycloheximide, permitting TNF to trigger apoptosis (Kreuz et al., 2001).

A different pathway for TNFR1-induced apoptosis critically depends on RIPK1, which is required for the activity of a death-inducing complex defined as complex IIb. This complex is composed of the proteins FADD, caspase-8 and RIPK1 (Wang et al., 2008). RIPK3 is likely also part of this complex, as it was shown to contribute to RIPK1-dependent apoptosis (Dondelinger et al., 2013). While RIPK1 has a scaffold function in TNFR1 complex I, which is independent of its kinase activity, its kinase activity is required for the activation of caspase-8 in TNFR1 complex IIb (Löder et al., 2012; Dondelinger et al., 2013). Therefore, the regulation of RIPK1 kinase activity, which induces its auto-phosphorylation on S166, is a critical checkpoint for TNF-induced apoptosis. In consequence, apoptosis controlled by this checkpoint can be prevented by RIPK1 inhibitors (Degterev et al., 2008). Moreover, provided that caspase-8 is inhibited, complex IIb can induce necroptosis. This also requires the kinase activity of RIPK1, which mediates the recruitment and activation of RIPK3 through the RHIM domains of both molecules (Cho et al., 2009; Li et al., 2012; Raju et al., 2018).

By default, upon TNFR1 stimulation, RIPK1 auto-activation is prevented by its ubiquitylation and phosphorylation (described in detail below). Thus, complex IIb induces cell death when, upon TNFR1 stimulation (i) the activity of E3 ligases such as the cIAPs and LUBAC is compromised or (ii.) the activity of RIPK1 inhibitory kinases, which are ubiquitylation-dependently recruited to and activated in the TNFR1-SC, is compromised (Figure 1B).

Accordingly, the loss of K63-linked polyubiquitylation upon TNFR1 activation results in RIPK1 dependent cell death. Treatment of cells with IAP inhibitors (SMAC mimetics, SM) results in K48-linked autoubiquitylation of cIAP1/2 and their rapid degradation (Petersen et al., 2007; Varfolomeev et al., 2007; Vince et al., 2007; Bertrand et al., 2008). In consequence, upon TNF/SM stimulation, RIPK1 ubiquitylation is reduced and RIPK1-dependent apoptosis ensues (Bertrand et al., 2008; Wang et al., 2008). In a physiological setting, cIAP1 can be degraded upon stimulation of the TNF-superfamily receptor FN14 by its ligand TNF-like weak inducer of apoptosis (TWEAK). Thereby, stimulation of FN14 and TNFR1 can cooperate to induce cell death which is blocked by RIPK1 inhibition (Vince et al., 2008; Wicovsky et al., 2009). Similar effects have been described with TNFR2, which, upon stimulation with transmembrane TNF, triggers the cytosolic depletion of TRAF2/cIAP1/2 and cooperates with TNFR1 to induce apoptosis (Chan and Lenardo, 2000; Fotin-Mleczek et al., 2002). In similarity to K63-linked ubiquitylation, M1-linked polyubiquitylation by LUBAC is crucial for the prevention of cell death by TNF. Mice deficient for the LUBAC component SHARPIN (*cpdm* mice) develop TNF-dependent dermatitis and multi-organ inflammation, which can be rescued by heterozygosity of caspase-8 or keratinocyte-specific loss of FADD, combined with loss of RIPK3, indicating cell death as the cause of inflammation (HogenEsch et al., 1993; Gerlach et al., 2011; Ikeda et al., 2011; Kumari et al., 2014; Rickard et al., 2014). This was shown to depend on the kinase activity of RIPK1, as RIPK^K45R^ knock-in *cpdm* mice were protected from multi-organ inflammation, and RIPK^K45R^ cells were shown to be protected from necroptosis (Berger et al., 2014). Similarly, MEF lacking HOIP were shown to undergo apoptosis upon stimulation with TNF, which partly depended on RIPK1 activity (Peltzer et al., 2014). Likewise, TNF induced cell death in absence of the LUBAC component HOIL-1, which was in part dependent on RIPK1 (Peltzer et al., 2018).

Consistent with the notion that RIPK1 ubiquitylation prevents TNF-induced RIPK1 activity and cell death, RIPK1 deubiquitylation by CYLD was reported to be required for apoptosis induction by complex IIb (Hitomi et al., 2008; Wang et al., 2008). Underscoring the cell-death promoting role of CYLD, the adaptor for the recruitment of CYLD to the TNFR1-SC, SPATA2, was shown to promote RIPK1-dependent apoptosis (Schlicher et al., 2016; Wei et al., 2017). Similarly, both CYLD and SPATA2 were reported to promote RIPK1 activation and TNF- induced necroptosis (Hitomi et al., 2008; Wang et al., 2008; Kupka et al., 2016; Wei et al., 2017). Counteracting its role in promoting necroptosis, CYLD was shown to be a substrate of non-apoptotic caspase-8 activity (O’Donnell et al., 2011).

The role of OTULIN in the disassembly of M1-linked polyubiquitin chains and the regulation of RIPK1-dependent death appears to be more complex. OTULIN specifically degrades M1-linked polyubiquitin chains, implying that it functionally counteracts LUBAC, thereby promoting TNF-induced cell death. However, a recent study suggests that the M1-linked auto-ubiquitylation of LUBAC inhibits its function, and decreases the abundance of LUBAC components. OTULIN, by deubiquitylating LUBAC, was suggested to promote LUBAC activity and thereby prevent the TNF-induced formation of complex II and cell death (Heger et al., 2018). Accordingly, fibroblasts derived from mice, which homozygously express an inducible, catalytically inactive OTULIN^C129A^ mutant, exhibited a substantial reduction of M1-linked polyubiquitin in the TNFR1-SC and enhanced formation of complex II and cell death upon treatment with TNF. The cell death was partly inhibited by RIPK1 inhibition, indicating that OTULIN activity maintains RIPK1-dependent and -independent pro-survival checkpoints. Consistently, the auto-inflammation in adult mice, which expressed inactive OTULIN, was dependent on cell death, as suggested by the finding that it was largely relieved by the combined loss of caspase-8 and RIPK3. This suggested that OTULIN, in similarity to LUBAC, prevents cell death (Heger et al., 2018). However, another study found that induced OTULIN deficiency in leukocytes did not result in cell death (Damgaard et al., 2016). Moreover, as cells expressing a patient-derived OTULIN^G218R^ mutant conferring pathologic inflammation were not sensitized to TNF-induced cell death, it is not clear how hypomorphic OTULIN mutations found in patients compare to OTULIN^C129A^ (Damgaard et al., 2019).

Together, K63-and M1-linked ubiquitylation in the TNFR1-SC is critical for the prevention of RIPK1-induced cell death. However, the specific requirement of RIPK1 ubiquitylation for the prevention of complex IIb formation was challenged by the finding that RIPK1 was ubiquitylated in complex II as well (Dondelinger et al., 2013; de Almagro et al., 2015). This raised the possibility that the restriction of RIPK1 activity depends on E3-ligases, but not on a direct effect of ubiquitylation on RIPK1.

Ultimately, ubiquitylation in the TNFR1-SC promotes the activity of kinases such as TAK1, IKKκ/β, or p38. Indeed, a number of recent studies showed that the inhibitory phosphorylation of RIPK1 by those ubiquitylation-dependent kinases prevents RIPK1 activity. A first indication came from the finding that TNFR1 ligation in the absence of TAK1 activity results in rapid apoptosis, which was dependent on RIPK1 kinase activity (Dondelinger et al., 2013). Interestingly, TNF-induced cell death upon cIAP inhibition could be reduced by knockdown of CYLD, while cell death by TAK1 inhibition was independent of CYLD, indicating that TAK1 represses RIPK1 activity downstream of ubiquitylation events. Indeed, NEMO/IKKα/β, the activity of which depends on TAK1, prevented RIPK1 activity and cell death independently of the induction of NF-κB (Legarda-Addison et al., 2009; Dondelinger et al., 2015). More recently it was demonstrated that IKKα/β phosphorylates S25 of RIPK1, thereby inhibitising its kinase activity. Accordingly, knock-in of a phospho-mimetic RIPK1^S25D^ mutant prevented RIPK1 auto-phosphorylation and cell death upon TNFR1 stimulation and IKK inhibition (Dondelinger et al., 2019). Underscoring the relevance of this phosphorylation, mice carrying the SHARPIN cpdm mutation, when crossed to RIPK1^S25D^ animals, were completely protected from multi-organ inflammation, in similarity to the protection provided by kinase dead RIPK1S45A (Berger et al., 2014).

In addition to IKK mediated phosphorylation, S321 and S336 of RIPK1 were shown to be phosphorylated in the cytosol by MK2, a downstream kinase of MAPK p38. While inactivation of MK2 by itself had no effect on TNF induced apoptosis, it further sensitized cells lacking IKK activity, or cells treated with SMAC mimetics, to TNF-induced RIPK1 activation, complex II formation and apoptosis (Dondelinger et al., 2017; Jaco et al., 2017; Menon et al., 2017). Consistently, stimulation of cells with TNF and TWEAK, resulting in the loss of TRAF2, reduced the activation of both IKK and MK2, permitting RIPK1 dependent cell death (Dondelinger et al., 2017).

Another study showed that the RIPK1 site shown to be targeted by MK2 by the studies above could be directly phosphorylated by TAK1 *in vitro*, however, the loss of S321 phosphorylation in cells lacking p38/MK2 activity possibly suggests that the effect of TAK1 on this site is mostly via its downstream kinase p38/MK2 (Dondelinger et al., 2015; Geng et al., 2017; Jaco et al., 2017; Menon et al., 2017).

More recently it was demonstrated that the kinases TBK1 and IKKε are, dependent on LUBAC-mediated M1-linked ubiquitylation and NEMO, recruited to the TNFR1-SC. While these kinases exhibited limited effects on TNF-induced gene expression, they were required to prevent TNF-induced cell death (Lafont et al., 2018; Xu et al., 2018). Direct phosphorylation of RIPK1 by TBK1/IKKε was suggested by RIPK1 gel shifts or the loss of phosphorylation of RIPK1S189 in cells upon TBK1/IKKε inhibition (Lafont et al., 2018; Xu et al., 2018). Furthermore, TNF-induced cell death of TBK1^-/-^ cells required RIPK1 kinase activity (Xu et al., 2018). Here, inhibition or loss of either TBK1 or IKKα/β (targeting different sites in RIPK1) permitted TNF-induced apoptosis, indicating that for prevention of RIPK1 mediated apoptosis, the simultaneous inhibitory phosphorylation of RIPK1 on different sites must be maintained (Lafont et al., 2018). Interestingly, IKKε had previously been reported to phosphorylate and inactivate CYLD, raising the possibility that the negative regulation of CYLD by IKKε also has a role in the prevention of RIPK1 dependent cell death in the context of inflammation as well (Hutti et al., 2009).

A recent study suggested that the prevention of RIPK1 activity by ubiquitylation does not necessarily depend on ubiquitylation-dependent kinases such as TAK1, IKK or p38/MK2. Instead, ubiquitylation, mediated by cIAP1, directly controlled RIPK1 activity. A point mutation in the UBA domain cIAP1, resulted in reduced interaction with TRAF2, but mediated otherwise normal NF-κB and MAPK activation and NIK degradation, indicating regular activation of the upstream kinases. Nevertheless, the reduced cIAP activity rendered cells more sensitive to TNF-induced cell death, due to reduced K48-linked polyubiquitylation of RIPK1, as well as reduced occupancy of lysines of RIPK1 by mono-ubiquitylation, which resulted in accumulation and increased (activating) auto-phosphorylation of RIPK1 (Annibaldi et al., 2018).

### Cell Death Induced by TLRs

While TNF-induced cell death has been subject to intense research, cell death induced by TLR signaling is much less investigated. MyD88-dependent TLR signaling does not directly induce cell death, however signaling through TRIF was shown to result in cell death by apoptosis which required FADD, caspase-8 and inhibition of the proteasome, presumably stabilizing IκB (Ruckdeschel et al., 2004). In another study, apoptosis upon TRIF overexpression was shown to require the RHIM domain of TRIF (Kaiser and Offermann, 2005). The involvement of ubiquitylation in the regulation of TRIF-dependent cell death was suggested by a study showing that poly (I:C) induced apoptosis was dependent on TLR3, TRIF, and caspase-8, which was counteracted by cIAP1 (Weber et al., 2010). Importantly, TLR3-induced apoptosis, promoted by the absence of cIAPs, required RIPK1 (Feoktistova et al., 2011; Estornes et al., 2012). One of these studies found active caspase-8 to be associated with the TLR3 (Estornes et al., 2012). In similarity to TNF-induced cell death signaling, upon inhibition of caspases, macrophages were shown to undergo necroptosis upon stimulation of TLR3 and TLR4. This was dependent on the presence of TRIF (He et al., 2011). In this study, RIPK1 knockdown resulted in macrophage death, where as Nec-1 prevented TLR3/4-induced necroptosis, indicating a prosurvival function of RIPK1, but the promotion of TRIF-induced cell death by its kinase activity. Another study on TLR-induced necroptosis confirmed the requirement for TRIF and RIPK1 kinase activity for macrophage necroptosis upon TLR3/4 stimulation and showed that macrophage necroptosis by stimulation of TLR2/5/9 required TNF to induce cell death. However, fibroblasts and endothelial cells did not require RIPK1 to undergo TLR3-induced necroptosis, suggesting a TRIF:RIPK3 complex to activate RIPK3 (Kaiser et al., 2013). This finding is supported by the contribution of TRIF to the perinatal RIPK3-dependent mortality of RIPK1^-/-^ mice (Dillon et al., 2014). Thus, unlike with TNF-dependent necroptosis, there is no absolute requirement for RIPK1 in TLR-induced necroptosis.

More recently, LUBAC was shown to prevent TLR3-induced apoptosis, as loss of HOIP or SHARPIN sensitized cells to cell death induced by poly (I:C) (Zinngrebe et al., 2016). This study identified the formation of a cytosolic death-inducing complex induced by TLR3, containing LUBAC, cIAP1/2, RIPK1, FADD, and caspase-8. Together, these reports demonstrate that K63-and M1-ubiquitylation represent pro-survival checkpoints not only in TNF-, but also in TLR-dependent cell death.

## Conclusion

The usual outcome of TLR and TNFR1 stimulation is pro-inflammatory gene expression, as different mechanisms or checkpoints prevent cell death upon stimulation of these receptors. In case of TNFR1 signaling, different levels of cell death prevention were defined, and interfering with these checkpoints [as often observed with pathogens (Lamkanfi and Dixit, 2010)] will reduce the threshold for cell death.

Cell death is prevented by TNFR1-induced gene expression, by transcriptional induction of pro-survival proteins. Another level of cell death suppression is the inhibition of the kinase activity of RIPK1, by the activity of E3 ligases such as cIAP1/2 and LUBAC, as well as kinases such as TAK, IKK, p38, and TBK1/IKKε. Interfering with these enzymes will, however, not only promote RIPK1 dependent cell death, but also inactivate the transcription-dependent checkpoint (with the exception of TBK1/IKKε, which have no role in pro-survival NF-κB induction by TNF (Pomerantz and Baltimore, 1999).

The actual and specific roles of cell death, triggered by the mechanisms described, remain to be investigated. It appears that, just as inflammatory gene expression triggered by innate immune and cytokine receptors, cell death induced via these receptors can likewise be beneficial or deleterious. Increasing evidence supports the concept that in addition to unrestricted pro-inflammatory signaling, the cell death-inducing activities of innate immune receptors have a key role for pathological inflammation. Cell death due to dysregulated ubiquitylation, as in LUBAC- or OTULIN defective animals or patients, can be a crucial trigger of pathologic chronic inflammation, with consequences such as auto-inflammatory disease (HogenEsch et al., 1993; Gerlach et al., 2011; Ikeda et al., 2011; Tokunaga et al., 2011; Berger et al., 2014; Kumari et al., 2014; Rickard et al., 2014; Boisson et al., 2015; Heger et al., 2018; Damgaard et al., 2019).

On the other hand, induction of cell death by apoptosis upon loss of ubiquitylation-dependent kinase activity has been shown to be beneficial. For example, the *Yersinia* protein YopJ, injected into target cells, blocks the kinase activity of TAK1 and thereby pro-inflammatory gene expression (Haase et al., 2005; Mukherjee et al., 2006). However, the resulting caspase-8 activation and cell death is in fact instrumental for defense against the pathogen (Philip et al., 2014; Weng et al., 2014). Accordingly, RIPK1^K45A^ (kinase dead) knock-in mice exhibited reduced macrophage cell death upon Yersinia infection, but succumbed rapidly to the infection (Peterson et al., 2017). Those examples likely reflect only an initial understanding of TLR- and TNFR1- induced cell death for both host defense and disease. Certainly, more work will be required to clarify the role of TLR- and TNFR1-induced cell death for limiting the spread of infection as well as causing human pathology.

## Author Contributions

LG generated the figures. All authors wrote the review.

## Conflict of Interest Statement

The authors declare that the research was conducted in the absence of any commercial or financial relationships that could be construed as a potential conflict of interest.

## Abbreviations

BMDM: bone marrow derived macrophages
CYLD: cylindromatosis
DUB: deubiquitinase
FADD: fas -associated protein with death domain
FLIP: flice inhibitory protein
HOIL-1: haem-oxidized IRP2 ubiquitin ligase-1
HOIP: HOIL-1 interacting protein
IAP: inhibitor of apoptosis
IKK: IκB kinase
IL: Interleukin
IRAK: interleukin-1 receptor associated kinase
LPS: lipopolysaccharide
LUBAC: linear ubiquitin chain assembly complex
MAPK: mitogen activated protein kinase
MK2: mitogen activated protein kinase-activated protein kinase 2
MKK: MAPK kinase
MLKL: mixed lineage kinase like
MLKL: mixed lineage kinase like
MyD88: Myeloid differentiation primary response 88
NEMO: NF-κB essential modulator
NF-κB: nuclear factor -κB
OTULIN: OTU deubiquitinase with M1-linkage specificity
SPATA2: spermatogenesis associated protein 2
PIM: PUB interacting motif
PUB: peptide:N-glycanase/UBA- or UBX-containing proteins
RIPK1: receptor interacting kinase 1
SC: signaling complex
SHARPIN: SHANK associated RH domain interactor
TAK1: TGF-beta activated kinase
TBK: TANK binding kinase
TBK1: TANK binding kinase
TLR: Toll-like receptor
TNF: tumor necrosis factor
NOD: Nucleotide-binding oligomerization domain-containing protein
TRADD: Tumor necrosis factor receptor type 1-associated death domain protein
UBA: ubiquitin associated
UBD: ubiquitin binding domain
